# Therapeutische Injektion und Manuelle Medizin beim tiefen Rückenschmerz: Bimodale Synergien zwischen Evidenz und Empirie

**DOI:** 10.1007/s00132-022-04235-8

**Published:** 2022-03-03

**Authors:** Uwe H. W. Schütz

**Affiliations:** 1 Schmerztherapie und Rheumatologie, Orthopädie am Grünen Turm, Grüner-Turm-Str. 4–10, 88212 Ravensburg, Deutschland; 2 Klinik für Diagnostische und Interventionelle Radiologie, Universitätsklinikum Ulm, Ulm, Deutschland

**Keywords:** Chronische Schmerzen, Injektion, Lumbago, Sakroiliakalgelenk, Wirbelsäule, Chronic pain, Injection, Lumbago, Sacroiliac joint, Spine

## Abstract

**Hintergrund:**

Angelehnt an die zielorientierte Therapieplanung und -führung in der Versorgung chronisch Rheumakranker, plädiert der Autor bei Patienten mit (chronischem) degenerativem tiefem Rückenschmerz (TRS), orientiert an den differenzialtherapeutischen Grundsätzen der Manuellen Medizin (MM), und unter Kenntnis von Techniken und Evidenzen therapeutischer Lokalinfiltrationen (TLI), für die Etablierung eines strukturierten mechanismenbasierten Therapiekonzeptes im Sinne des „treat to target“ (T2T) im ambulanten schmerztherapeutischen Versorgungsbereich.

**Diagnostik:**

Dies setzt eine konsequente (Primär‑)Diagnostik mit Schmerzanalyse unter der Prämisse, dass TRS, wenn strukturell-funktionell bedingt, immer spezifisch ist, voraus. Eine breite biopsychosoziale Anamnese und strukturbezogene klinisch-bildgebende (Ausschluss‑)Diagnostik mit funktioneller Differenzierung nach MM-Grundsätzen und ggf. interventionelle Blocks, sollten am Ende zur Formulierung einer 3‑Ebenen-Diagnose als Voraussetzung zu einer mechanismenbasiert-zielorientiert-hierarchischen Stufentherapie bei TRS führen. Diese wird in diesem Artikel pragmatisch fallorientiert, unter Implementierung von Techniken und Evidenzen der TLI und MM, vorgestellt.

## Abstract (en)

**Background:**

Oriented towards the therapy planning and management of rheumatic patients, and based on the differentiated therapeutic principles of manual medicine (MM) with knowledge on evidence of therapeutic local infiltration techniques (TLI), the author pleads for the establishment of a structured, mechanism-based therapy concept in the sense of “treat to target” (T2T) for patients with (chronic) degenerative low-back pain (LBP) in outpatient pain therapy care.

**Diagnostics:**

This requires a consistent (primary) diagnosis with pain analysis under the premise that LBP is always specific if it is structurally and functionally conditioned. A broad bio-psycho-social anamnesis and structure-based clinical diagnosis (imaging) with functional differentiation according to MM principles and, if necessary, interventional blocks, should result in the expression of a three-level diagnosis as a prerequisite for a mechanism-based, hierarchic step therapy in LBP. In this article, this is presented in a pragmatic, case-oriented manner, with the implementation of techniques and evidence of TLI and MM.

Die Kenntnis der Mechanismen der Schmerzphysiologie sowie die Beherrschung der Methoden der Manuellen Medizin und der therapeutischen Lokalinterventionen, ermöglicht dem ärztlichen Therapeuten, nach suffizienter Diagnostik der primären Schmerzgeneratoren, einen mechanismenbasierten, stadiengerechten synergistisch sinnvollen bi-/multimodalen Methodeneinsatz zur gezielten Behandlung akuter und chronischer, spezifischer tiefer Rückenschmerzen. Am Ende der Behandlung kann eine relevante anhaltende Schmerzreduktion als Voraussetzung für die Rekonditionierung der neuromotorischen Systeme stehen.

## Einleitung

Neben der medikamentösen Systemtherapie sind die Techniken zur Durchführung therapeutischer Lokalinfiltrationen (TLI) und die Methoden der Manuellen Medizin (MM) die wichtigsten Werkzeuge zur Behandlung des tiefen Rückenschmerzes (TRS) in der ambulanten Versorgung. Sie werden von Ärzten verschiedener Fachrichtungen angewandt (z. B. Orthopäden und Unfallchirurgen, Spezielle Schmerztherapeuten, Neurologen, Anästhesisten, Allgemeinmediziner, Rheumatologen).

In der Schulmedizin ist die Stellung der korrekten Diagnose Voraussetzung für eine suffiziente Behandlung des TRS. Hierbei geht es in der Praxis jedoch nicht um die simple Auswahl einer annähernd passenden Diagnose aus dem ICD-Katalog, sondern um die Formulierung einer Funktionsdiagnose, welche die individuell vorliegende Ursache der TRS des Patienten wiedergibt. Damit dies dem Behandler überhaupt gelingen kann, muss dieser die Mechanismen der Schmerzentstehung und -verarbeitung auf Basis eines vertieften Kenntnisstandes hinsichtlich funktioneller Neuroanatomie und -physiologie kennen und verstanden haben.

Anhand eines klinischen Fallbeispiels soll in diesem Artikel dem Leser das auch für den ausgebildeten Facharzt durchaus sehr anspruchsvolle Ziel der Überführung der sehr theorielastigen, neurophysiologischen Erkenntnisse zum TRS (seien sie nun akut oder chronisch, unspezifisch [11] oder spezifisch [34]) in eine alltagstaugliche ursachenbasierte Funktionsdiagnose aufgezeigt werden, auf der erst dann die Etablierung und zielorientierte stadiengerechte mechanismenbasierte (Schmerz‑)Therapie bzw. Auswahl und Kombination von Verfahrensweisen der TLI und MM möglich wird.

### Tiefer Rückenschmerz

#### Neuroanatomie

Bei der neuroanatomischen Betrachtung der Lumbosakroiliakalregion (LSIR) fällt eine hohe Innervations- und Nozizeptordichte der (peri-)vertebralen Strukturen auf [40]. Die verschiedenen Gewebe weisen, mit Ausnahme des Nucleus pulposus, allesamt nozizeptive Fasern auf, welche unter adäquater Reizung (thermisch, mechanisch etc.) Erregungen des nozizeptiven Systems generieren (Tab. 1). Ist der Nucleus pulposus als größtes avaskuläres humanes Gewebe primär kein Ort möglicher Nozizeption, so ändert sich dies aufgrund einsetzender Neovaskularisation, sobald es zu einer Bandscheibenschädigung gekommen ist [21], was die hohe Rezidivrate bzw. Vulnerabilität bezüglich des TRS bei vorliegenden Diskopathien mit erklären mag.

**Table 1:** 

Noziregion	Physiologisch	Pathologisch
Ossär	Rr. communicantes Nn. spinales Gefäße (vegetativ, sympathisch)	Subkortikale Hypervaskularisation bei Diskopathie
Ligamentär	Ligg. ileolumbale, Ligg. longitudinale etc. Intraspinal: z. B. Ligg. flava	Überdehnung, Zerrung Enthesiopathie, Fasziitis Hypertrophie
Diskogen	Anulus fibrosus	Diskopathie/Diszitis: Neovaskularisation des N. pulposus
Neural (Dura)	Partiell	Neuritis
Arthrogen (FG, SIG)	Gelenkkapsel Mit Meniskoiden (bei > 50 % vorhanden), partiell vaskularisiert und innerviert	Arthritis, Kapsulitis, Synovialitis Einklemmung von Meniskoiden: Einblutung, Degeneration
Myofaszial	Myofaszialer Nervenfaserverlauf	Propriozeptive Rückkopplung: Muskelhypertonus/Myalgie Myositis, Fasziitis

*FG* Facettengelenk, *Ligg.* Ligamente, *SIG* Kreuzdarmbeingelenk

#### Neurophysiologische Mechanismen der Schmerzentstehung und -verarbeitung

In den letzten beiden Dekaden haben die konsequenten translationalen Bemühungen von manualmedizinischen (Schweizerische Ärzte- bzw. Deutsche Gesellschaft für Manuelle Medizin SAMM/DGMM) und schmerztherapeutischen Ärztegesellschaften (IGOST: Interdisziplinäre Gesellschaft für orthopädische/unfallchirurgische und allgemeine Schmerztherapie) zur Zusammenführung der Erkenntnisse der neurophysiologischen Grundlagen und der klinischen Schmerzforschung zu einem vertieften Verständnis der Mechanismen der Schmerzentstehung und -verarbeitung geführt, auf welche sich auch u. a. die moderne MM stützt. Zum Verständnis des vorliegenden Artikels werden die diesbezüglich etablierten Fachbegriffe und zugrundeliegenden Mechanismen vorausgesetzt, welche in der einschlägigen Fachliteratur zum chronischen TRS [52, 66, 75, 81] und auch im Beitrag H. Schnell et al. dieses Themenheftes („Die segmentale und somatische Dysfunktion. Wie funktioniert Manuelle Medizin?“) als Erklärungsmodell der Wirkungsweise der MM auf neurophysiologischer Ebene, aufgezeigt werden. Diese ineinandergreifenden Mechanismen umfassen von der einfachen akuten Nozizeptoraktivierung bis hin zur komplex-chronischen globalen Hyperalgesie u. a.:

- (nozireaktive) motorische Systemaktivierung (Nozireaktion/-reflex)
- periphere Sensibilisierung, neurogene Inflammation, primäre Hyperalgesie
- afferente Konvergenz via multirezeptivem Hinterhornneuron (WDR-Neuron „wide dynamic range neuron“ = spinothalamisches Konvergenzneuron)
- sympathische Systemaktivierung, parasympathische Dysregulation
- zentrale Sensibilisierung (spinaler „crosstalk“, „primping“), neurogene Neuroinflammation
- Aktivierung rezeptiver Felder, „referred pain“, „wide spread pain“
- sekundäre Hyperalgesie
- Dysregulation schmerzinhibitorischer Systeme
- zentrale Chronifizierung, Schmerzkrankheit

Da die neurogene Inflammation, als relativ früher einsetzender Mechanismus der peripheren Sensibilisierung, auf isolierte MM-Interventionen nicht anspricht, dienen primär antiinflammatorische Analgetika als spezifische Therapeutika.

#### Injektable analgetisch-antiinflammatorische Agenzien

 Therapeutische Lokalanästhesie (TLA) unter Verwendung niedrigdosierter Lokalanästhetika (LA) werden seit mehr als 10 Dekaden zur Reduktion der Nervenerregbarkeit bzw. Schmerzen (Blockade sensibilisierter Nervenfasern) und Verbesserung der Durchblutung eingesetzt [48]. Unter anderem werden dabei die dünnen sensiblen Schmerzfasern (C-Fasern und Aδ-Fasern) weitgehend blockiert, wobei die Erregbarkeit der dickeren motorischen Fasern i. d. R. erhalten bleibt. Mechanismen der desensibilisierenden Wirkung der LA sind direkt anti-inflammatorische LA-Effekte und die Reduktion der „neurogenen Inflammation“ durch verminderte Ausschüttung von Neuropeptiden (z. B. CGRP, Substanz P), welche die Prostaglandin- und Zytokinbildung (z. B. TNF-α, IL-6, IL-1ß) fördern [13]. Additiv beigefügte Glukokortikoide (GK) können den pathologischen Nozizeptorschmerz aufgrund des inflammatorisch gereizten Gewebes (neurogene Inflammation), sowie auch einen neuropathischen Schmerz (inflammatorisch gereizte Nervenfasern), d. h. auch den „mixed pain“, effektiv down-regulieren [4]. Durch Reduktion der Synthese von Interleukinen und Phospholipase A2 und durch Vasoprotektion wirken GK hemmend auf den, v. a. durch cytokinproduzierende Zellen ausgelösten, biochemischen Entzündungsprozess bei der Radikulitis ein: Hemmung von Migration bzw. Ausdifferenzierung von Entzündungszellen zu Makrophagen [67]: TLA-Wiederholungen verbessern den Wirkeffekt bzw. wirken durch eine verlängerte Desensibilisierung der Schmerzchronifizierung entgegen [46].

### Initialdiagnostik

Die First-Line-Diagnostik bei TRS umfasst neben der allgemeinen und vegetativen (Fremd‑)Anamnese die spezifische Schmerzanamnese, welche die Evaluierung von Schmerzlokalisation, -qualität, -quantität (VAS/NRS), -häufigkeit, -dauer sowie der internen Trigger- und Risikofaktoren („red flags“ [102]) umfassen sollte. Bei V. a. chronischen TRS ist die validierte Evaluation bzgl. Chronifizierungsstadium (MPSS [32]) und neuropathischer Schmerzkomponente („pain detect“ [31]), wie auch die anamnestische Erfassung psychosozialer beruflicher („blue/black flags“ [42]) und soziofamiliärer („yellow flags“ [11]) Risikofaktoren der Schmerzchronifizierung Standard.

Die physikalische Untersuchung ist das zentrale Element der diagnostischen Bemühungen

Die physikalische Untersuchung als zentrales Element der diagnostischen Bemühungen umfasst dabei klassischerweise die Inspektion von Gang, Achssymmetrie, Haltung etc., die Palpation hinsichtlich Dermographismus, vegetative Zeichen, Gewebeturgor etc., und die funktionelle aktive/passive Bewegungsprüfung (ROM) mit spezifischen Funktionstests. Orientierende neurologische Untersuchung hinsichtlich Durchblutung, Motorik und Sensibilität, Reflexstatus, Nervendehnungs-/-provokationszeichen etc. sind inbegriffen.

Additiv ist für den manualmedizinisch tätigen Arzt die segmentale nozizeptive Funktionsanalyse mittels 3‑Schritt-Diagnostik (MIP) zur segmentalen Evaluierung von *M*obilität (segmental), *I*rritation (nozireaktiver Muskelhypertonus) und *P*rovokation (Reversibilität?) zentrales und einziges gut reliables, europäisch konsentiertes und definiertes, manualdiagnostisches Instrument [92, 101] zum Einstieg in mechanismenbasierte Überlegungen.

#### Bildgebung

Bei akutem TRS (unter 6 Wochen Schmerzdauer nach Erstauftreten) ist eine Bildgebung dann sicher indiziert, wenn aufgrund Anamnese und klinischer Untersuchung Hinweise für einen spezifischen Rückenschmerz oder „red flags“ bestehen [11]. Laut Leitlinie ist für die Diagnose eines spezifischen Kreuzschmerzes i. d. R. eine bildgebende Diagnostik notwendig [34]. Eine wiederholte analgetisch-antiphlogistische Medikation und/oder MM-Therapie muss also nach Verstreichen der Akutphase der TRS bei ausbleibender Beschwerdelinderung hinsichtlich deren Ursache spezifiziert bzw. bildgebend abgeklärt werden. Die Basisdiagnostik besteht aus einem konventionellen Röntgenbild der (LWS im Stand in 2 Ebenen) sowie einer Magnetresonanztomographie (MRT) der LWS [34], eventuell auch der Sakroiliakalgelenke (SIG), unter Berücksichtigung des § 23 RöV und der Leitlinien der Bundesärztekammer (BÄK) zur Qualitätssicherung in der Röntgendiagnostik [50]. Die Diskussion, ob Röntgen oder MRT, ist nicht neu und richtet sich nach der Aussagefähigkeit der Untersuchung im Hinblick auf die Verdachtsdiagnose [86], aber initial auch nach der Verfügbarkeit, der Frage der Strahlenexposition und der Kosten [11]. Daher wird die Frage einer notwendigen Computertomographie (CT) der LSIR nur bei traumatischen oder präoperativen Fragestellungen relevant [86]. Bezogen auf die MM *in der Akutphase von TRS*, ist nach Durchführung einer Probemobilisation vor einer Manipulation ein vorheriges routinemäßiges LWS-Röntgen nicht notwendig [11, 43].

#### 3-Ebenen-Diagnose

In der Regel sollte zu diesem Zeitpunkt eine erste mechanismenbasierte Funktionsdiagnose angestrebt werden. Hier hat sich in den letzten Jahren in der MM (DGMM) die Formulierung der sog. 3‑Ebenen-Diagnose als praktikables und sinnvolles Tool erwiesen [52], welches auf der ersten *Ebene A* die Symptombeschreibung der Beschwerden des Patienten und dessen subjektives Erleben, sowie die zeitlichen (Akuität, Chronizität) und räumlichen Faktoren (Lokalität) beinhaltet. Die zweite *Ebene B* umfasst die Beschreibung der möglichen somatischen Genese der Beschwerden, einschließlich struktureller und funktioneller Befunde, sowie, falls bereits möglich, die mechanistische Genese mit Fokus auf den neurophysiologisch-mechanistischen Faktoren. Zuletzt folgt *Ebene C* mit der Berücksichtigung der soziofamiliären Situation des Patienten (Beruf, Familie, Ökonomie).

#### Schmerzmuster

Aus dem Schmerzmuster („pain mapping“) kutanomyofaszialer Schmerzen, welches sich bei Patienten mit TRS im Rahmen der klinisch-palpatorischen Diagnostik evaluieren lässt, kann der erfahrene Untersucher wertvolle Rückschlüsse hinsichtlich der Schmerzgenese ziehen [37, 63, 103]. Entscheidend ist hierbei, die fokalen Schmerzen und myofaszialen Triggerpunkte von ausstrahlenden, fortgeleiteten und übertragenen Schmerzen richtig zu differenzieren und einzuordnen (Tab. 2).

**Table 2:** 

Schmerzmuster	Schmerzcharakteristik (Symptomatik/Klinik)	Neurophysiologische Schmerzmechanismen
Lokaler Schmerz	Fokale somatische Schmerzen über dem Schmerzgenerator, evtl. mit Hyperalgesie/-ästhesie z. B. Arthritis/aktivierte Arthrose über FG/SIG; Enthesiopathie: z. B. SIPS, Myalgie (z. B. M. lumborum)	*Primäre Hyperalgesie:* akut, nozizeptiv neurogene Inflammation
Übertragener Schmerz („referred pain“)	Schmerzprojektion aus tiefen viszeralen (o. somatischen) Strukturen auf ein konstantes Segment „Stechender“, durchdringend, brennender u./o. temperaturempfindlicher Schmerz z. B. Hyperalgetische Zonen: Head-Zonen (Haut) oder McKenzie-Zonen (Muskeln) z. B. Muskelhypertonie, autonome Veränderungen (keine Reflexalteration/Muskelatrophie)	Afferente Konvergenz aus Neurotom-Viszerotom-Myotom-Dermatom auf die Hinterhornzelle (WDR)
Myofasziale Triggerpunkte	Schmerzprojektion auf nahe bis überregionale Areale in tiefe anatomische Schichten (myofaszial) „Nackter-einfacher“ Schmerz z. B. pseudoradikulärer Schmerz als Ausdruck artikulärer Störung mit reflektorischer Muskelirritation (wirbelsäulennaher Schmerzgenerator)	Langsame zentrale Sensibilisierung und Ausbreitung auf rezeptive Felder [63] *sekundäre Hyperalgesie* (neuropathisches Geschehen), Senkung Schmerzschwelle
Ausstrahlender, fortgeleiteter Schmerz	Vor allem im Ausbreitungsgebiet neuraler Strukturen (Dermatom, Myotom, Sklerotom) z. B. Neuralgie, Plexusneuritis, Radikulopathie	Neuritis (neurale Inflammation)

*FG* Facettengelenk, *SIG* Kreuzdarmbeingelenk,* SIPS* Spina iliaca posterior superior, *WDR* „wide dynamic range neuron“

### Kasuistik-Teil 1: Klinische Kasuistik

Ein 55-jähriger Mann, der sich initial mit seit 3 Tagen exazerbierten TRS (lumbal bilateral gluteal bis in den dorsolateraleren Oberschenkel rechts reichend) mit einer Schmerzstärke von 9 (10-Pkt.-VAS) vorstellt.

#### Anamnese

Seit ca. 12 Monaten bestehender „stechend-ziehender“ Schmerz in der rechtsdominanten LSIR, rezidivierend bis zur laterodorsalen Unterschenkelregion reichend. Hierbei nur kurze schmerzfreie Intervalle über 1–3 Tage bei einer Schmerzdauer von mehreren Tagen bis Wochen. Belastungsabhängige Schmerzverstärkung am Tage, bewegungs- und lagerungsabhängiger Schmerz in Ruhe und zur Nacht und v. a. morgens beim Aufstehen.

Nebenerkrankungen: Diabetes mellitus Typ IIb (Rp Metformin), arterielle Hypertonie (Rp Ramipril), Z. n. Koronarstents bei 2‑Gefäß-KHK, Hypercholesterinämie (Rp Statin), Adipositas (BMI 33 kg/m^2^), Obstipationsneigung mit gelegentlich Unterbauchschmerzen.

Sozialanamnese: Identifikation mit und Freude am Beruf als Altenkrankenpfleger (2-Schicht-System mit regelhaften Transferarbeiten). Soziofamilär seit Jahren zufrieden verheiratet, 2 erwachsene Kinder (außer Haus). Tennis als Hobby, seit Monaten schmerzbedingt pausiert.

#### Klinischer Erstbefund

**Figure 1:**
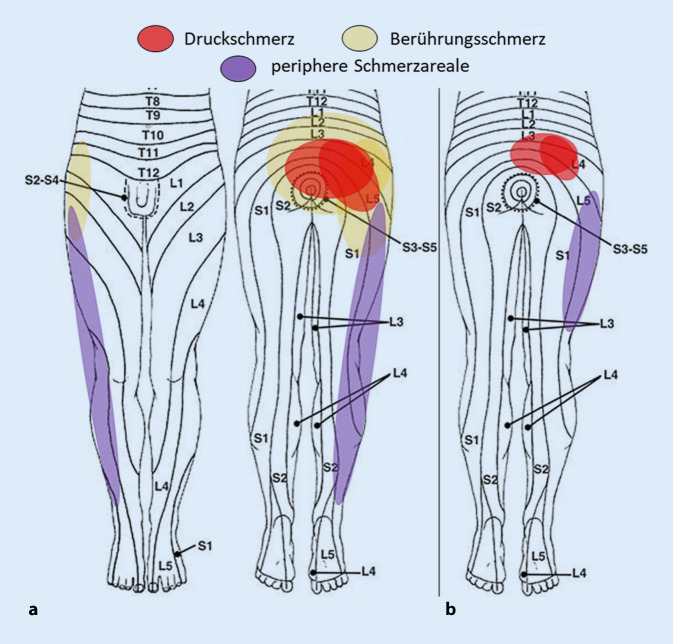

Schmerzbedingte linksorientierte Rumpfschiefhaltung mit Entlordosierung der LWS, deutlicher Reduktion des Finger-Boden-Abstands und LWS-Entfaltungsstörung (Reduktion Schober/Ott). Segmentale Funktionsanalyse (MIP) schmerzbedingt kaum möglich bzw. nicht interpretierbar. Orientierend neurologisch unauffällige Sensibilität, (Pseudo‑)Lasègue ab 60° links, beidseits symmetrische Hyporeflexie bzgl. Achillessehnenreflex/Patellarsehnenreflex. Palpatorisches Schmerzmuster mit ausgeprägter Berührungsempfindlichkeit der gesamten lumbopelvinen Region bis gluteal und starke Druckempfindlichkeit lumbosakral bilateral paravertebral (L3-S2) und über den SIPS und proximale SIG bis gluteal links (Valleix-Punkt links positiv). Die Betrachtung der entsprechenden „pain map“ in Abb. 1a und b führt differenzialdiagnostisch nicht sicher weiter, da vereinbar mit (Tab. 2):

- lokalem Schmerz bei Facettensyndrom (FGS) L5/S1 (oder L4/5) [73, 108] bei Spondylarthrose,
- viszerogen übertragenem Schmerz („referred pain“): z. B. aus Dickdarm (Th11), bei bekannten Unterbauchschmerzen [103],
- pseudoradikulärem Schmerz als Ausdruck artikulärer Störung mit reflektorischer Muskelirritation (manifestiert als Triggerpunkte) von M. iliocostalis/longissimus [28], M. quadratus lumborum oder M. gluteus medius [24], und/oder
- radikulärem Schmerz entsprechend Dermatom L5/S1 rechts, bei Minderung des Intervertebralraumes (IVR) L5/S1.

#### Röntgen LWS in 2 Ebenen (Abb. 2)

Orthogerader Aufbau (a.-p.) und Entlordosierung (Seitprojektion), Osteochondrose L5/S1 (Grad 3–4) bei Diskopathie (IVR-Höhenminderung 70–90 %) und Spondylarthrose L5/S1 (Grad 2–3) und L4/5 (Grad 2). Partiell abgebildete kaudale SIG-Arthrose links (Grad 2–3).

**Figure 2:**
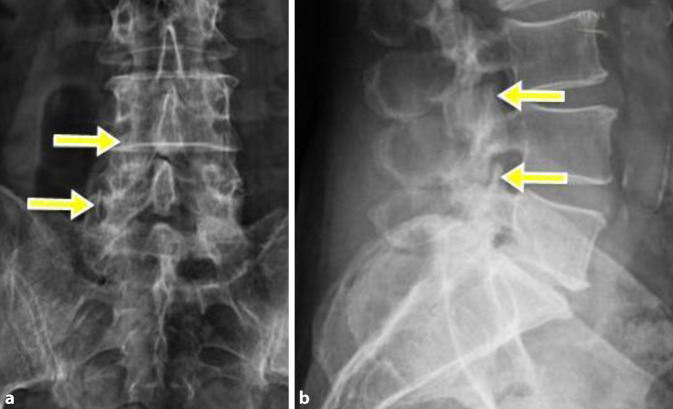

#### Procedere

Bei dem Patienten findet sich übersichtradiographisch eine fortgeschrittene IVR-Degeneration als Hinweis auf eine relevante Diskopathie, klinisch ist eine biomechanisch bedingte Wurzelirritation L5/S1 links möglich. Dies bedingt aufgrund der Ausprägung der Beschwerdesymptomatik die weiterführende Abklärung mittels MRT der LWS und Einholung einer neuro(physio)logischen Mitbeurteilung.

### Multiple Schmerzgeneratoren bei degenerativem TRS

Eine progrediente IVR-Degeneration führt bei Verlaufsprogredienz biomechanisch, pathophysiologisch bedingt zu multilokulären Veränderungen mit möglicher Veränderung und/oder Verschlimmerung der Symptomatik, auch über das Segment hinaus.

Bei der alters- bzw. be-/überlastungsbedingten Bandscheibendegeneration, welche i. d. R. im Bereich L3-S1 aufsteigend initiiert wird, kommt es durch Dehydrierung des Bandscheibengewebes u. a. zu kleinen Einrissen mit Strukturschwäche des Anulus fibrosus und konsekutiver Vorwölbung, welche favorisiert dorsal betont imponiert und hier auch fokal zum Riss des Anulus fibrosus mit Austritt von Nucleus-pulposus-Material führen kann. Diese Zeichen der degenerativen Diskopathie sind nur mittels MRT der LWS direkt darstellbar („black disc“, Protrusion, Prolaps) [86].

Mit zunehmender Höhenminderung der Bandscheibe kommt es zur Annäherung der entsprechenden Wirbelkörper (röntgenologisch indirekt durch die Reduktion der IVR-Höhe detektierbar: Chondrose), was auch zu einer Erhöhung der Druckbelastung der angrenzenden Boden- bzw. Deckplatten führt, welche mit der Zeit zur Strukturveränderung der Kortikalis führt (Röntgen: Sklerose). In der Progredienz kommt es am IVR zur Bildung ossäre Abstützreaktionen (Röntgen/MRT: Spondylophyten), welche regelhaft anterolateral, aber auch dorsal auftreten können. Im Verlauf kommt es zu einem fettig-degenerativen Umbau (MRT: Typ Modic 2) und/oder Dehydrierung des angrenzenden Markraumes (MRT: Typ Modic 3). Bei diesem Bild der Osteochondrose findet sich, als i. d. R. stärker beschwerdebehaftetes Zwischenstadium, eine entzündliche Überlastungsreaktion des angrenzenden spongiösen Markraumes mit primär intrakorporaler Ödembildung (MRT: Typ Modic 1), welches dann als erosive Osteochondrose bezeichnet wird.

Die IVR-Höhenminderung führt auch zu vermehrten chondralen Druckbelastungen in den Facettengelenken (FG). Hierdurch wird ein Arthroseprozess initiiert, wie er auch pathophysiologisch an peripheren Gelenken auftritt [85]. Es entsteht eine Spondylarthrose, welche in Progredienz typische bildgebende Arthrosezeichen entwickelt: Inkongruenz der Gelenkpartner, FG-Hypertrophie durch Osteophytosis, Ergussbildung. So kommt es auch bei weiterer Progredienz von Degeneration und Höhenminderung des IVR zur Deformierung von angrenzenden Deck- und Bodenplatten bzw. Wirbelkörperanteilen, auch mit partieller Gefügelockerung, was eine segmentale Stellungsveränderung der Wirbelkörper bedingen kann, welche entweder als Retro- oder Pseudolisthese oder/und multisegmental zu eine sog. De-novo-Skoliose der LWS führen kann.

Retrospondylophytosis, Verkalkung des hinteren Längsbandes, reaktive Hypertrophie der Ligg. flava, Listhese, Skoliose und/oder Diskusprotrusion/-prolaps können eine segmentale Stenosierung neuraler Räume bedingen, welche dann in der MRT als spinale, rezessuale und/oder intraforaminale Stenosen auf osteo-, disko- und/oder ligamentärer Basis zu beschreiben sind und als Radikulopathie und/oder Claudicatio spinalis im Sinne des neuropathischen Schmerzes bzw. Geschehens symptomatisch werden.

**Figure 3:**
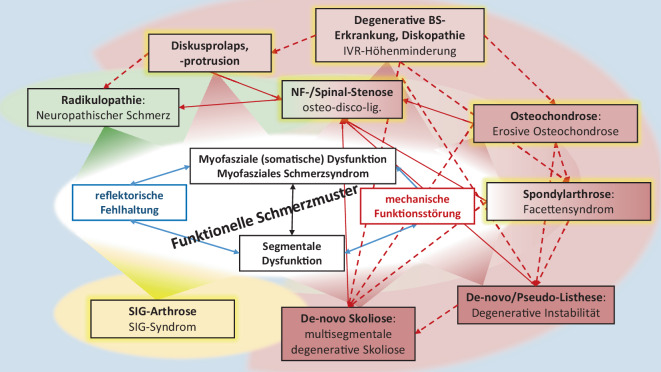

Die beschriebene Strukturpathogenese progredienter degenerativer Segmentveränderungen, welche allesamt mittels MRT (Goldstandard beim TRS) detektierbar sind [86], werden in ihrem komplexen gegenseitigen Bedingungsgefüge in Abb. 3 (roter Bereich) grob schematisch aufgezeigt.

Unter Kenntnis der möglichen Schmerzgeneratoren (Tab. 1) und der Neurophysiologie der Schmerzentstehung und -weiterleitung [52, 66, 75, 81], können alle in Abb. 3 aufgezeigten Strukturpathologien (rot-grün-gelber „Ring“) primärer Schmerzgenerator bei degenerativem TRS sein. Wie aufgezeigt, kann jeder dieser primären Schmerzgeneratoren schmerzreflektorisch (segmentale Nozireaktion) funktionelle Schmerzmuster (Abb. 3: zentral weiß hervorgehoben) verursachen, welche klinisch als myofasziales Schmerzsyndrom/Dysfunktion und/oder hypomobile segmentale Dysfunktion (Blockierung) zum Ausdruck kommen.

## Manuelle Medizin bei TRS: Definition, Indikation, Evidenz

Eine einheitliche Definition der Manuellen Medizin gibt es nicht. Laut (Muster‑)Weiterbildungsordnung der BÄK [107] umfasst sie „… die Erkennung und Behandlung reversibler Funktionsstörungen des Bewegungssystems einschließlich ihrer Wechselwirkung mit anderen Organsystemen mittels manueller Untersuchungs- und Behandlungstechniken.“

Im Kern bedient sich die MM der Manipulation und der Mobilisation. Erstere sind Techniken mit Impuls und werden im Bereich der Osteopathie als HVLA-Techniken („high velocitiy low amplitude“) bezeichnet und haben beim TRS ihre Indikation bei reversiblen, hypomobilen Funktionsstörungen ohne Radikulopathie (= Blockierung) in der LSIR [11, 51], welche leitlinienkonform Ursache spezifischer TRS sein kann [34]. Techniken der Mobilisation umfassen beim TRS u. a. wiederholt, rhythmisch-federnde Traktions-Translations-Bewegungen, z. B. Muskel-Energie-Techniken, und im osteopathischen Bereich auch Strain‑/Counterstrain, Myofascial Release etc., und haben ihre Indikationen in allen reflektorischen und teils strukturellen Dysfunktions- und Schmerzzustände der LSIR mit (pseudo‑)radikulärer Ausstrahlung verschiedenster Genese [11, 51], zu der v. a. das myofasziale Lumbalsyndrom (Dysfunktion) als Ursache spezifischen TRS [34] zählt. Hinsichtlich der schmerzreduzierenden Wirkungsweise der MM wird auf die einschlägige Fachliteratur [51, 54] und auf den Beitrag von H. Schnell et al. in diesem Themenheft („Die segmentale und somatische Dysfunktion. Wie funktioniert Manuelle Medizin?“) verwiesen.

### Evidenz der MM

Wie aus Tab. 3 ersichtlich, ist bei Anlegung von evidenzbasierten Kriterien die Effektivität für die Manipulation beim akuten und chronischen TRS nur mäßig, für die Mobilisationstechniken eher sogar schlecht. Lediglich eine Selbstbewertung kommt zu einer positiveren Bewertung, welche keiner Evidenzkontrolle standhält [65], zumal selbst führende Kapazitäten auf dem Gebiet der MM deren Evidenzlage als nur mäßig [53] bewerten.

**Table 3:** 

TRS	Manipulation	Mobilisation (Muskel-Energie-Techniken etc.)
Akut	Paige et al., Metaanalyse, 2017; 15 RCT [70]: *mittel bis -mäßig* (−10 VAS) Franke et al., Metaanalyse, 2014 [29], HVLA: *mäßig* (−13 VAS)	Orrock, Review, 2015 [68]: *schlecht* (nicht besser als „sham“)
	Cochrane-Reviews, 2012-15; 4 RCT [30, 79] „akuter nicht-spezifischer TRS“: *schlecht* (nicht besser als „sham“, auch nicht in mit Bewegungstherapie)	
Selbstbewertung:	Locher et Moll, 2017 [53]: *mäßig* Niemer, 2015 [65]: *„positiv“*	–
Chronisch	Cochrane-Review, 2011; 9 RCT [8]: *gering bis nicht relevant* Franke et al., Metaanalyse, 2014 [29], HVLA: *mäßig* (−15 VAS)	Cochrane-Review, 2015 [68]: *schlecht* (nicht besser als „sham“)
	NVL-Empfehlung, 2017 [11] „nicht-spezifischer TRS“: kann empfohlen werden (Empfehlungsgrad *„offen“* *=* *0*)	
Selbstbewertung:	Locher et Moll, 2017 [53]: *mäßig* Niemer, 2015 [65]: *„positiv“*	Niemer, 2015 [65]: *mäßig*

*HVLA* „high velocity low amplitude“, manipulative Methode der Osteopathie, *NVL* Nationale Versorgungsleitlinie, *RCT* randomisierte kontrollierte Studie, „*sham“* Placebo, *TRS* Tiefer Rückenschmerz, *VAS* 100-Punkte Visuelle Analogskala

Betrachtet man obige Ausführungen zu den vielfältigen Möglichkeiten der primären Schmerzgenerierung beim TRS und dessen strukturpathologisches Bedingungsgefüge bis hin zur regelhaften Folgegenerierung funktioneller Schmerzmuster (Abb. 3), so gibt dies einen Hinweis, dass ein myofasziales Lumbalsyndrom und eine Blockierung in Gegenwart degenerativer Veränderungen zu einem hohen Anteil sekundärer Natur und nicht regelhaft primäre Entitäten sind. In der Schlussfolgerung ist dann die schlechte Evidenzlage der Therapiewirkung von MM auch mit dadurch erklärbar, da oft der primäre Schmerzgenerator (Strukturpathologie) und damit der Verursacher funktioneller Störmuster bei Therapie noch gar nicht identifiziert bzw. eliminiert ist, was konsekutiv Rezidive von myofaszialer und hypomobiler somatischer Dysfunktionen in der LSIR trotz wiederholter MM-Interventionen erklärt.

Insofern muss jeder MM-Therapeut bei, trotz lege artis durchgeführter MM-Techniken, rezidivierend auftretenden funktionellen Schmerzmustern ein kritisches Re-Assessment durchführen und prüfen, ob die Diagnostik hinsichtlich der Detektierung des primären Schmerzgenerators abgeschlossen oder auszuweiten ist, bzw. sich fragen, ob er den Patienten in seinem TRS mechanismenbasiert korrekt erfasst hat.

### Kasuistik-Teil 2: 3-Ebenen-Diagnose und Erstbehandlung

Nach Erstdiagnostik wird folgende Funktionsdiagnose gestellt:

*Ebene A:* Exazerbiertes rechtsdominantes Schmerzsyndrom der LSIR bei chronischer belastungs- und bewegungsabhängiger Lumbosakralgie mit rezidivierendem Beinschmerz rechts dorsolateral.

*Ebene B:*

- chronisches (sekundäres) myofasziales Schmerzsyndrom mit segmentaler Funktionsstörung (Blockierung)
- Strukturpathologien: Osteochondrose L5/S1, Spondylarthrose L4-S1, SIG-Arthrose links
- DD Schmerzmuster: (Pseudo‑)Radikulopathie, „referred“ (viszeral: Colon?), lokal (FG?, SIG?)
- exazerbierter Schmerz (neurogene Neuroinflammation, primäre Hyperalgesie, motorische Systemaktivierung, periphere Sensibilisierung)
- chronischer Schmerz, „wide spread pain“ (sekundäre Hyperalgesie, zentrale Sensibilisierung)

*Ebene C:*

- „black flag“: Transferarbeiten. Freude am Beruf (Ausschluss „blue flags“)
- soziofamiliär, psychogen keine Auffälligkeiten (Ausschluss „yellow flags“)

**Figure 4:**
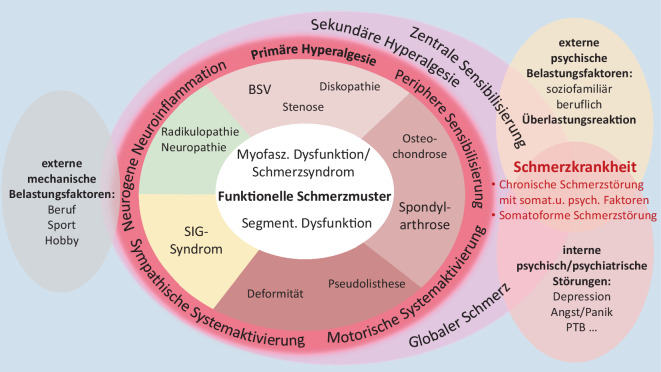

Aktuell ist eine erneute MM-Therapie aufgrund der Schmerzexazerbierung nicht durchführbar und auch nicht indiziert, da sie bisher nur sehr kurzfristig zur Besserung betrug. Die Chronifizierung lässt vermuten, dass, bei vorliegender primärer Hyperalgesie bei peripherer Sensibilisierung, neurogener Neuroinflammation sowie sympathischer und motorischer Systemaktivierung, der Übergang zur sekundären Hyperalgesie mit zentraler Sensibilisierung und damit zum zentralen Chronifizierungsprozess bereits begonnen hat – auch wenn bei dem Patienten „nur“ externe mechanische Belastungsfaktoren im Beruf („black flags“) und keine externen psychischen soziofamiliären („yellow flags“) oder beruflichen („blue flags“) und auch keine intrinsischen psychisch/psychiatrischen Störungen vorliegen (Abb. 4).

In der vorliegenden klinischen Situation hat die rasche und wirksame Schmerzreduktion oberste Priorität, um eine klinische Feindiagnostik und auch die Therapiefähigkeit bezüglich konservativ multimodaler Interventionen zu ermöglichen. Nach dem Prinzip „hit hard and early“ ist hier eine Methodik zu wählen, die rasch und effektiv ist, sodass der Kaudalblock eine Möglichkeit darstellt, um dieses Ziel zu erreichen. Bei Beherrschung und korrekter technischer Durchführung dieser Technik, bietet dieser gegenüber der potenten Schmerzinfusion den Vorteil der guten Verträglichkeit ohne relevante systemische Belastung.

Beim Patienten wurde bei Erstvorstellung ein landmarkengestützter Kaudalblock (Gemisch: Triamcinolon 20 mg Off Label, 15 ml Ropivacain 2 mg/ml) durchgeführt (Technik siehe in [47, 98], bildgestützt in [41]). Gleichzeitig zur Desensibilisierung bei Hyperalgesie der apikalen LSIR, wie man sie bei exazerbiertem TRS häufig sieht (Triggerpunkt: SIPS), wurde eine spezielle landmarkenorientierte fächerförmige Infiltrationstechnik in den dorsalen Bandapparat der SIG und an den Ansätzen des Lig. iliolumbale durchgeführt (Gemisch: je Seite Triamcinolon 10 mg, 5 ml Mepivacain 0,5 %, 6–8 cm Kanüle), was einer „semi-targeted“ fokal/regional weichteilbasierten TLI bzw. proximal periartikulären SIG-Infiltration entspricht (Tab. 5**, **Technik siehe in [47, 98, 100]).

## Therapeutische Lokalinterventionen beim TRS: Techniken, Effektivität, Evidenz

Bei chronischem TRS ist nach Evaluation durch ein ausgedehntes Expertengremiums die Evidenz für 62 % der diagnostischen und 52 % der therapeutischen Interventionen moderat (angemessen) bis gut [58]. Grob orientierend lassen sich die perkutanen minimal-invasiv-interventionellen Verfahren zur mehr oder weniger gezielten Schmerztherapie beim TRS wie in Tab. 4 dargestellt einteilen. Betrachtet man die TLI-Techniken an der LSIR im Speziellen, so besteht ein Unterschied in den therapeutischen Evidenzen.

**Table 4:** 

	Methodik
*Fokal/regional weichteilbasierte TLI* „semi-/untargeted“ (nur landmarkenorientiert^a^)	*Neuraltherapeutische Injektionen/Infiltrationen:*
	Triggerpunkt-gestützte myofasziale oder
	Leitmuskel-basierte, DAVOS-orientierte myotendinöse TLI
	SIPS-Region-Infiltration (nozizeptorreiche Ansatzregion der lumbosakroiliakalen Bänder), ist keine typ. SIG-Injektion
	*Prolotherapie*
	*Botox-Injektionen*
	*s.c.-Infiltrationen (Quaddelung)*
	*Ungezielte i.m. Injektionen (meist gluteal), systemischer Ansatz*
*Wirbelsäulennahe TLI* „targeted“ (mit oder ohne Bildunterstützung^a,b,c,d^)	*Epidurale Injektionstechniken (EPI):*
	Kaudal‑/Sakralblock^**a,b,c,d**^
	Transforaminal via Foramen sacrale 1^**a,b**^ (Technik des Autors)
	Interlaminär dorsal (LOR bei Landmarkentechnik)^**a,c,d**^
	Interlaminär rezessual-perineural^**a,c,d,e**^
	Extraforaminal (= LSPA)^**a,b,d,e**^
	Transforaminal lumbal (= PRT)^**d**^
	*Facettengelenksinterventionen (FGI):*
	Perikapsulär^**a,b,c**^
	i.a./transkapsulär^**c,d,e**^
	„Medial-branch“-Block^**c,d,e**^
	*Sakroiliakalgelenk (SIG):*
	Periartikulär^**a,b,c**^
	i.a./transkapsulär^**a,b,c,d,e**^
	*Intradiskale Injektion *^***c,d,e***^
*Denervationen* *Ablationen * (nur bildgestützt^c,d^)	*FG-Denervation/Ablation *^***c,d,f***^*: RF-Neurotomie (konventionell-bipolar-cooled-gepulst). Thermokoagulation, Kryosonden, YAG-Laser*
	*SIG-Ablation (i.a. oder „lateral branches“) *^***c,d***^
	*SCS: „spinal cord stimulation“*
	*Intrathekale Opioidgabe*
	*Intradiskale Ablation*

*BV* Bildverstärker/Bildwandler (z. B. C‑Bogen), *CT* Computertomographie, *DAVOS* „da wo’s weh tut“, *EPI* epidurale Injektion, *FGI* Intervention am Facettengelenk, *i.a.* intraartikulär, *i.m.* intramuskulär, *LOR* „lost of resistance“-Technik (landmarkenorientiert ohne Bildunterstützung), *LSPA* lumbale Spinalnervanalgesie, *PRT* periradikuläre Therapie, *RF* Radiofrequenz, *s.c.* subkutan, *SIG* Sakroiliakalgelenk, *SIPS* Spina iliaca posterior superior, *Sono* Sonographie/Ultraschall

^a^landmarkenorientiert

^b^sonographisch gestützt

^c^Bildwandler-gestützt (z. B. C‑Bogen)

^d^CT-gestützt (nur mit Fachkunde CT)

^e^MRT-gestützt (klinisch noch nicht richtig etabliert)

Es ist u. a. der Verdienst der ASIPP, die anhand systematischer Literaturreviews der zurückliegenden knapp 40 Jahre (von 1966 bis 2011) hinsichtlich der Betrachtung der Effektivität im Jahr 2012 „Licht ins Dunkel“ der Evidenz bildgestützter lumbal, para- und periduraler LA-GK-Injektionen gebracht hat (Tab. 5). Das geschah unter Anlage hoher Assessmentqualität und klinischer Relevanzkriterien (u. a. Cochrane-Musculoskeletal-Review-Group-Kriterien für randomisierte Trials bei interventionellen Techniken, Newcastle-Ottawa-Scale-Kriterien für fluoroskopische Beobachtungsstudien, Evidenzqualität: U.S. Preventive Services Task Force). Dabei wurden das primäre Outcome-Kriterium Schmerzreduktion (in Kurzeit < 6 Mon. und Langzeit > 6 Mon.) und sekundäre Kriterien wie Verbesserung von Funktionsstatus, psychologischem Status und Arbeitsfähigkeit, sowie Reduktion der Opiatsubstitution berücksichtigt.

**Table 5:** 

Schmerzgenerator	Injektionstechnik 1	Injektionstechnik 2	Injektionstechnik 3
Therapeutische epidurale Injektionen (EPI)			
Technik:	*Kaudale EPI („kaudaler Block“)*	*Interlaminäre EPI*	*Transforaminale Injektion (PRT)*
*Reviews gesamt:* *(nur bildgestützt, Ausnahme LOR)*	LA + GK: *gut* [1, 72], *überlegen* [74], *positiv* [77] nur LA: *moderat* [72]	LA + GK: *gut* [6, 105], *moderat* (nur KZ) [16, 77], (LOR-Technik: *schlecht* [71]) nur LA: *moderat* [6] nur GK: *schlecht* [7, 8], *moderat* [44]	LA + GK: *gut* [5, 10, 60, 74], *moderat* [61, 78], Operationsvermeidung [76, 78] nur LA: *moderat* [16, 60, 76] nur GK: *schlecht* [76]
*ASIPP-Reviews, 1966–2011* *(nur bildgestützt):*	[72] *inkludiert: 16, RCT: 11, non-RCT (mit BV) 5* revidiert: 145 von 653	[6] *inkludiert: 26, RCT.:23* revidiert: 150 von 520	[10, 60] *inkludiert: 25, RCT: 15* revidiert: 117 von 428
BSV und/oder Radikulopathie	LA + GK: *gut* LZ & KZ nur LA: *moderat*	LA + GK: *gut* LZ & KZ nur LA: *moderat*	LA + GK: *gut* LZ & KZ nur LA: *moderat*
Diskogener oder axialer TRS	LA + GK:* gut*	LA + GK: *gut* nur LA: *schlech***t**	LA + GK: *schlecht* nur LA: *schlecht*
Spinalstenose	LA + GK: *moderat*	LA + GK: *moderat*	LA + GK: *moderat*
„Post-surgery“-Syndrom	LA + GK: *moderat*	*?*	LA + GK: *schlecht* nur LA: *schlecht*
Therapeutische lumbale Facettengelenksinterventionen (FGI)			
Technik:	*Intraartikuläre FGI*	*„Medial-branch“-Block*	*Radiofrequenzablation*
*Reviews gesamt:* *(nur bildgestützt, Ausnahme LOR)*	*Schlecht* [15, 26, 62, 96, 108]	LZ: *gut* [26], LZ: *moderat* [9, 15, 23, 62, 96, 108] KZ: *gut* [62, 108], *moderat* [9]	*Gut* [26], *moderat* [15, 62, 96], (*gepulst limitiert*, 1 non-RCT)
*ASIPP-Review, 1966–2011 [*26*]* *inkludiert: 25, RCT: 11, non-RCT 14* revidiert: 122 von 335	*Positiv*: 5 non-RCT *negativ*: 2 RCT, 1 non-RCT	LA + GK/nur LA: *positiv* LZ: 1 RCT, KZ: 1 RCT	*Positiv***,** LZ: 6 RCT LZ, 7 non-RCT *negativ*: 1 RCT

*ASIPP* American Society of Interventional Pain Physicians, *BSV* Bandscheibenvorfall, *BV* Bildverstärker, *EPI* epidurale Injektion, *FGB* Facettengelenkblock, *FGI* Intervention am Facettengelenk, *GK* Glukokortikoid, *LA* Lokalanästhetikum, *KZ* Kurzeit (Wirkung bis 6 Monate), *LOR* „lost of resistance“-Technik (landmarkenorientiert ohne Bildunterstützung), *LZ* Langzeit (Wirkung > 6 Mon.), *PRT* periradikuläre Therapie, *RCT* randomisiert kontrollierte Studie

Hinsichtlich der nichtbildgestützten diesbezüglichen Injektionen, sog. landmarkengestützte Injektionen, können (wie bei der MM auch) bis heute in der Literatur keine Studien oder Reviews ausreichender Qualität detektiert werden, die eine Metaanalyse zulassen oder hinreichend für eine Evidenzevaluierung der therapeutischen Wirksamkeit wären. Wie bei der MM, besteht auch für die landmarkengestützten Injektionen hinsichtlich der Therapieeffektivität jedoch eine jahrzehntelange positive klinische Empirie bei gleichzeitig hoher Praktikabilität und Umsetzbarkeit in der Praxis am Patienten. Auch wenn diese evidenzbasierten Kriterien nicht standhalten, sind sie aus der v. a. ambulanten Versorgung des TRS nicht wegzudenken bzw. hier unentbehrlich.

Nahezu alle etablierten, zielorientierten bildgestützten wirbelsäulennahen Injektionen [39, 41, 47] sind auch landmarkenorientiert ohne Bildunterstützung durchführbar. Voraussetzung zur Anwendung muss aber sein, dass der Anwender diese Techniken, wie bei der MM auch, beherrscht. Hier reicht es nicht aus, sich die, v. a. auf Krämer et al. [45, 90] basierenden Landmarkentechniken autodidaktisch anhand der speziellen Fachliteratur [47, 98–100] anzueignen. Es ist eine qualitätskontrollierte Schulung notwendig, um dieses Verfahren sicher und gezielt am Patienten indikationsorientiert anwenden zu können. Daher werden diesbezüglich Kurse der entsprechenden Fachgesellschaften angeboten (z. B. strukturiertes Aufbaucurriculum der IGOST). Sie sollten absolviert werden, da sie zunehmend zur Voraussetzung für die Abrechenbarkeit entsprechender Leistungen werden.

### Neuraltherapeutische Infiltrationen und Injektionen beim TRS

Dies sind triggerpunktgestützte myofasziale oder leitmuskelbasierte, evtl. auch DAVOS-orientierte („da wo’s weh tut“) myotendinöse TLI, die als Instrument in der ambulanten Versorgung des lokalen und pseudoradikulären Lumbalsyndroms und des SIG-Syndroms mit oder ohne Blockierung etabliertes Instrument sind. Interessanterweise gibt es auch hier keine hinreichenden Evidenzen in der Literatur, es mangelt an entsprechenden Studien guter Qualität. Typische Zielregionen der Infiltration sind die dorsolateralen Crista iliaca, SIPS-Region und paraspinös segmentale Regionen.

### Epidurale Injektionen (EPI) beim TRS

Die lumbosakrale epidurale Injektion (EPI), ist die häufigste Interventionen zur Therapie starker überregionaler TRS. Ihre Evidenz variiert je nach Techniken stark ([72]; Tab. 5). In ihrer Subgruppenanalyse evaluierte die ASIPP nur für die Indikationsgruppe lumbaler BSV und/oder Radikulopathie eine gute Evidenz für alle 3 EPI-Verfahren (kaudaler Block, interlaminäre EPI und transforaminale EPI), wenn neben LA zusätzlich ein GK beigemischt wird; ohne GK bei nur LA-EPI besteht diesbezüglich eine moderate Evidenz. Verwendet man ein LA-GK-Gemisch, so ist die transforaminale EPI nur noch bei der Spinalstenose von moderater Evidenz; bzgl. diskogenem, axialem Schmerz und bei „Post-surgery“-Syndromen ist diese nicht empfohlen (schlechte Effektivität). Interlaminäre EPI und Kaudalblock mit LA-GK-Gemisch zeigen bei diskogenem, axialem Schmerz und Spinalstenose eine moderate Effektivität.

#### Off-Label

Bezüglich der EPI-Techniken muss erwähnt werden, dass bei perineuralen Injektionen die GK nicht zugelassen sind (Ausnahme: CT-gestützte PRT bei Triam-Lichtenstein). Dies steht im Gegensatz zur evidenzbasiert aufgezeigten Notwendigkeit der Zugabe von GK zum LA, um einen guten Therapieeffekt zu erreichen. Dies bedeutet, dass jegliche EPI mit GK einer Off-Label-Aufklärung und -Einwilligung durch den Patienten bedarf und nicht auf Basis der gesetzlichen Krankenversicherung abgerechnet werden darf.

#### Strahlenschutz

Hinsichtlich der bildgestützten Interventionen wird derzeit (noch) an der Wirbelsäule und am SIG die Verwendung strahlenintensiver Verfahren, v. a. Bildverstärker/-wandler (BV, oft in Form des C‑Bogens) oder CT, favorisiert. Viele Techniken sind jedoch auch sonographisch gestützt machbar und beschrieben [56, 97]. Der MRT obliegt hier nach Auffassung des Autors in naher Zukunft, bei fehlender Strahlenbelastung und zunehmend etablierter Real-Time-Protokolle, die Rolle des Goldstandards. Bis dahin ist die Voraussetzung zur Vermeidung überhöhter Strahlenbelastung die Kenntnis der Regeln des Strahlenschutzes und der Technik des verwendeten Verfahrens. Beim BV spielt die Durchleuchtungszeit eine zentrale Rolle, was nur durch Routine bzw. gute Schulung erreicht werden kann, um die „learning curve“ möglichst kurz zu halten [87, 89]. Was die CT betrifft, so ist die fachgerechte Nutzung der „Low-dose“-CT unabdingbar, um nicht nur den Behandler, sondern auch den Patienten vor einer unverhältnismäßig hohen Strahlenbelastung zu schützen. Wer also mit dosisrelevanten Begriffen, wie „shadowing, overbeaming, overranging, pitch, windowing“, Filterkern, Rekonstruktionsalgorithmus, Schichtkollimation und SNR, nichts anfangen kann, muss die Hände von CT-gestützten Interventionen lassen, wenn kein Radiologe unterstützend zur Seite steht [83]. Nur so kann die „Low-dose“-CT-gestützte Injektion mit 0,25 mSv ähnlich niedrige Strahlungsexpositionen wie die gepulste Röntgenfluoroskopie (ca. 1,0 mSv) gewährleisten [2, 14, 82, 84].

#### Kontraindikationen

Kontraindikationen (v. a. bei EPI-Techniken) wie „red flags“, Medikamentenunverträglichkeiten, lokale trophische Störungen, lokale/systemische Infekte, Leukopenie, ZNS-Erkrankungen, schwere Herz-Kreislauf-Erkrankungen und Gerinnungsstörungen sind sorgfältig auszuschließen. Aufgrund möglicher (sehr seltener) schwerwiegender Komplikationen, sollte v. a. bei EPI-Techniken die direkte Notfallversorgung bis hin zum ACLS („advanced cardiac life support“) gewährleistet sein [13]. Bei Einnahme antithrombotisch wirksamer Medikamente ist die aktuelle DGAI-Leitlinie „Rückenmarksnahe Regionalanästhesien und Thromboembolieprophylaxe/antithrombotische Medikation“ [106] eine gute Orientierung. INR/Quick und Thrombozytenzahlen sollten Mindestwerte von 1,2/70 % bzw. 100.000/μl haben.

### Kasuistik-Teil 3: Re-Assessment nach 10 Tagen

#### MRT LWS, nativ

**Figure 5:**
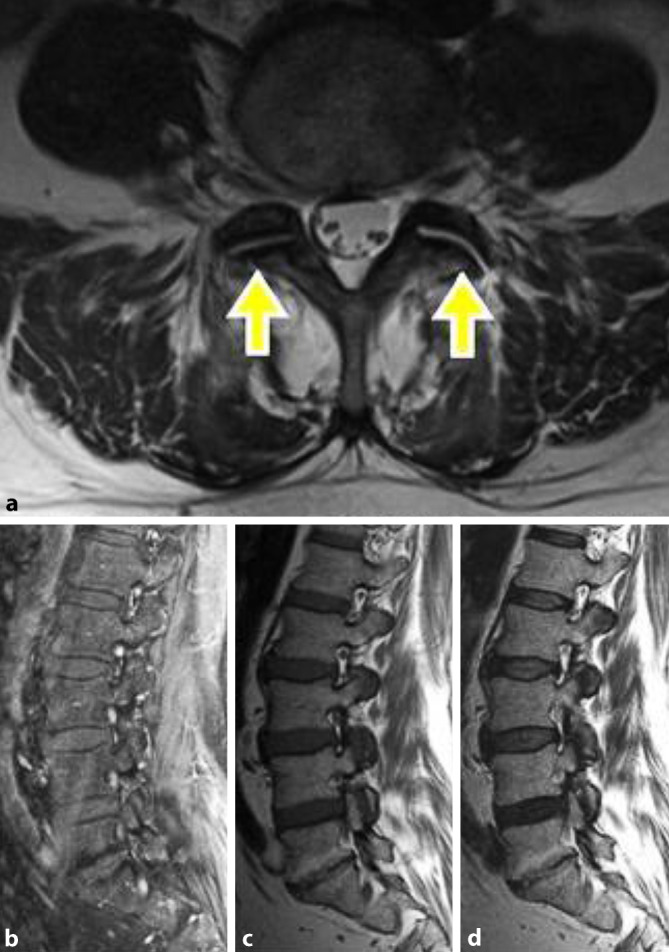

Im Segment L5/S1 Nachweis einer fettig degenerativen Osteochondrose (Modic-Typ 2) und Spondylarthrose beidseits mit i.a. Flüssigkeit links. Fettiger Umbau der medianen tiefen paraspinalen Muskulatur, keine SIG-Signalalteration (Abb. 5**)**.

#### Klinisches Bild

Neurologie: Unauffällige Neurophysiologie für L5 und S1, Ausschluss einer Radikulopathie.

Im klinische Re-Assessment nach kaudaler EPI und Neuraltherapie SIPS beidseits deutliche Beschwerdebesserung:

- gerader Stand, keine Schmerzfehlhaltung, Hyperlordose LWS
- keine Berührungsempfindlichkeit mehr, noch deutliche Druckempfindlichkeit lumbosakral bilateral FG L5-S1 bds., SIPS links, gluteal links (Valleix negativ)
- MIP: keine freie Richtung, Muskelhartspann M. lumborum bds. und gluteal links
- Lasègue negativ, Sensibilität o.B., Hyporeflexie Achillessehnenreflex/Patellarsehnenreflex bds. unverändert symmetrisch
- Schmerzmuster: typisch für FG-Syndrom lumbosakral rechtsdominant (Abb. 1b)
- typischer FG-Federungsschmerz [20, 59]

Bei klinisch bildgebend hochgradigem V. a. auf Vorliegen eines aktivierten FG-Syndroms L5/S1 bei chronisch degenerativem Lumbosakralsyndrom wurde der Patient über die Möglichkeit der Durchführung eines FG-Denervation/Ablation nach Diagnosesicherung über einen diagnostischen FG-Doppelblock L5/S1 informiert. Nach Aufklärung über die Funktionsbeeinträchtigung der segmentalen Mm. multifidii durch Denervation der Rr. dorsales [94], lehnte der Patient diese Maßnahmen ab und stimmte alternativ der beidseitigen GF-Infiltration L5/S1 zu, welche BV-gestützt (je FG, Gemisch: Triamcinolon 40 mg, 1 ml, 10 ml Mepivacain 0,5 %) 3‑malig im Abstand von 7 Tagen leitlinienkonform durchgeführt wurde [19, 23, 34, 58].

### Facettengelenkinterventionen (FGI) beim TRS

Seit Schwarzer et al. [91] gilt, dass es keine validen klinischen Kriterien bzw. keine klinischen Funktionstest gibt, welche geeignet sind, die Diagnose eines lumbalen FGS sicher zu verifizieren. Der (nichtfunktionellen) medizinischen Bildgebung (Röntgen, CT, MRT) kommt hierbei „nur“ die Rolle der Ausschlussdiagnostik zu, da auch eine hierdurch detektierte starke Spondylarthrose klinisch dauerhaft symptomlos sein kann (Reliabilität und prädiktiver Wert sind gering) [80]. Die angegebene Spannbreite hoher Prävalenzen des FGS (36–67 %) als Ursache chronifizierter TRS ist aufgrund der hohen Wahrscheinlichkeit eines positiven Testergebnisses für die Präsenz der Pathologie lumbales FG-Syndrom erklärt [3, 25], und man geht daher davon aus, dass dieses weniger häufig als Ursache spezifischer Kreuzschmerzen vorliegt als diagnostiziert.

Die Diagnose eines FGS als spezifischer TRS ist auf Basis der Daten der ASIPP-Research-Group ([27]: *n* = 325, gesichtet 170, implementiert 23) nur dann gut valide, wenn dies durch einen Doppelblock, also eine 2‑malige Durchführung eines bildgestützten FG-Blockes mit unterschiedlich lang wirksamen LA, unter einem Outcome-Parameter von mind. 75 % Schmerzreduktion eruiert wird. Doppelblocks mit Outcome 50–74 % Schmerzreduktion sind, ebenso wie Kontrollblocks mit Placebo, in ihrer diagnostischen Aussagekraft moderat bis schlecht [88], also nicht indiziert. Einzelblocks sind als diagnostisches Verfahren obsolet, da schlecht in ihrer Evidenz [27].

Betrachtet man die therapeutischen FGI, so sind intraartikuläre FG-Injektionen, egal ob mit oder ohne GK, in ihrer Effektivität hinsichtlich der Schmerzreduktion als schlecht zu bewerten, wohingegen der „Medial-branch“-Block bei serieller Durchführung (3- bis 6‑malig) bzgl. der Kurz- und Langzeitwirkung gute Evidenzen aufzeigt. Gleiches gilt für die Radiofrequenzneurotomie (RFN) des lumbalen FG (Tab. 5), wobei die gepulste RFN hier nur limitierte Ergebnisse aufzeigt. Diese Daten beziehen sich durchweg auf bild-, meist röntgengestützte Interventionen, bzgl. Sonographie‑, CT- und landmarkengestützte Verfahren liegen keine suffizienten Studiendaten oder Evidenzangaben vor.

### Interventionen am Sakroiliakalgelenk beim TRS

Die Rolle des SIG-Syndroms beim TRS ist intensiv erforscht. Die Innervation des SIG ist komplex und, wie seine Größe und Form (Heterogenität), individuell sehr variabel [18]. Dadurch kann das SIG als Schmerzgenerator im klinischen Bild (SIG-Syndrom), wie ein Chamäleon, durch unterschiedlichste Schmerzmuster in der LSIR bis in die Oberschenkel und die Leisten reichend imponieren [38]. Daher kann, ähnlich wie beim FGS, weder die Anamnese noch die klinisch-funktionelle Untersuchung und/oder ein radiologisches Merkmal die Diagnose SIG-verursachter Schmerz sicherstellen. Dies erklärt, warum die Prävalenz von SIG-Schmerzen je Studiendesign breit zwischen 10 und 62 % streut. Die Mehrheit der von Simopoulos et al. [93] analysierten Studien von 1966–2012 (*n* = 2736, gesichtet 450, 18 implementiert), deutet auf eine Punktprävalenz von ca. 25 % hin.

Eine diagnostische SIG-Blockade kann nur durch sichere i.a.-Injektion ins SIG durchgeführt werden, was nur bildgestützt (BV, CT, MRT) möglich ist. Bildgestützt durchgeführte unkontrollierte SIG-Blocks zeigen eine falsch-positive Rate von ca. 20 %. Hingegen ist, wie auch beim FGS, die Evidenz für den kontrollierte SIG-Doppelblock (vergleichende LA-Blockaden bei Outcome-Kriterium mindestens 70 % Schmerzreduktion) im systematischen Review von Simopoulos et al. [93] gut, die für provokative diagnostische Tests nur mäßig.

Therapeutisch werden intra- und periartikuläre Injektionen, Sakralastblöcke und HF-Ablation, sowohl landmarken- als auch bildgestützt, verwendet, um Patienten mit SIG-Schmerzen zu helfen ([95], Tab. 4). Diese interventionsbasierte Therapie des SIG-Syndroms ist durch eine große Variabilität und einen Mangel an qualitativ hochwertigen Studien gekennzeichnet, sodass hier keine validen Ergebnisse eruierbar sind. Die vorliegenden klinischen Studien beschreiben einen mittelfristigen Nutzen sowohl für intra- als auch extraartikuläre GK-Injektionen [38]. Bei Patienten, die keine anhaltende Linderung durch SIG-Injektionen erfahren, kann die RF-Denervation eventuell eine Linderung von bis zu 1 Jahr bewirken.

### Sonstige interventionelle Verfahren beim TRS

#### Prolotherapie, Botulinumtoxin (Btx)

Auf die Verfahren der Prolotherapie (hyperosmolare Dextrose) und der Btx-Injektionen kann hier hinsichtlich Durchführung, Wirkungsweise, Effekt und Evidenz nicht näher eingegangen werden. Für diese Therapieformen besteht für den TRS keine Evidenz, da es an entsprechenden Studien mangelt bzw. die Fallzahlen zu gering sind [95]. Im Bereich der lumbalen FG und der paravertebralen Muskulatur werden sie als eher ineffektiv beschrieben [17, 33]. Metaanalysen sind bis dato nicht möglich [104]. Einzelstudien bzgl. dem SIG sind vielversprechend: Eine prospektive Fall-Kontroll-Studie von Lee und Kollegen [49], welche die BV-gestützte periartikuläre Btx-SIG-Injektion mit der KG-LA-Injektion verglich, kam in der die klinische Wirksamkeit zu dem Ergebnis, dass bei der Schmerzreduktion im 1. postinterventionellen Monat kein Unterschied bestand, jedoch in der Btx-Gruppe in der Nachbeobachtung nach 2 und 3 Monaten im Vergleich signifikant anhielt. Auf die entsprechende Fachliteratur wird verwiesen [49, 50, 95].

#### Intradiskale Interventionen (IDT)

Die intradiskalen Interventionen (IDT) mit GK und PRP („platteled enriched plasma“) sind in den Hintergrund geraten, da die nur kurzfristigen positiven schmerzhemmenden Effekte den vergleichsweise hohen Prozedurenaufwand im Ergebnis nicht rechtfertigen [22, 64]. Die intradiskalen Ablationen bewirken oftmals aufgrund der iatrogenen Gewebeschädigung einen Progress der Diskopathie mit all deren das gesamte Segment betreffenden möglichen Folgen (s. oben). Die IDT mit Ozon (O_2_/O_3_) scheint bei monosegmentaler Diskopathie vergleichsweise positive Effekte bzw. Empfehlungsgrade zu haben [35, 57, 69].

### Kasuistik-Teil 4: Re-Assessment, 4. Woche

#### Klinisches Bild

Subjektiv noch diskreter residueller bewegungsabhängiger Schmerz lumbosakral links:

- diskrete Druckempfindlichkeit lumbosakral bilateral über FG L5/S1 links (kein FG-Federungsschmerz)
- MIP: freie Richtung (L4 + li, lo), Muskelhartspann M. lumborum links

#### Procedere

Manipulation (L4 re, ky):

- Krankengymnastik: entlordosierend-stabilisierend zur Anleitung („Rückenschule“)
- Ergotherapie: Ergonomie am Arbeitsplatz
- Akupunktur: Regulation inhibitorischer Systeme, Desensibilisierung

#### Weiter Verlauf in 2 Jahren

- noch 2‑malig funktionelle Lumbosakralgie auf niedrigem Niveau
- „back to work“ 6 Wochen nach Erstvorstellung, keine weitere Arbeitsunfähigkeit: innerbetrieblicher Bereichswechsel (keine regelhaften Transferarbeiten mehr)
- Einleitung hausärztlich supervidierte Gewichtsreduktion
- Tennis moderat

## Zusammenfassung

Ein *primäres* funktionelles lumbosakrales Schmerzmuster beim degenerativen TRS ist, wenn keine Kontraindikationen vorliegen, eine Indikation für eine initiale direkte MM-Behandlung, da in diesem Falle die Blockierung und/oder die myofasziale Dysfunktion die Ursache spezifischer TRS ist, da primärer Schmerzgenerator. Ist das funktionelle Schmerzmuster jedoch Folge strukturell-degenerativer Prozesse, so ist das funktionelle Schmerzmuster *unspezifisch*, da sich der primäre Schmerzgenerator auf strukturpathologischer Ebene befindet und somit Ursache spezifischer TRS ist (z. B. degeneratives FGS, erosive Osteochondrose). Diese Differenzierung wird in der Leitlinie „Spezifischer Kreuzschmerz“ [11] jedoch nicht getroffen, was in der Weiterentwicklung nachzuholen sein wird. Insofern kann man die Kritiker der in derzeitiger Form vorliegenden S2K-Leitlinie verstehen, wenn sie die funktionellen Schmerzmuster bei der Diagnose „Nichtspezifischer Kreuzschmerz“ [52] weiter angesiedelt sehen (wollen).

**Figure 6:**
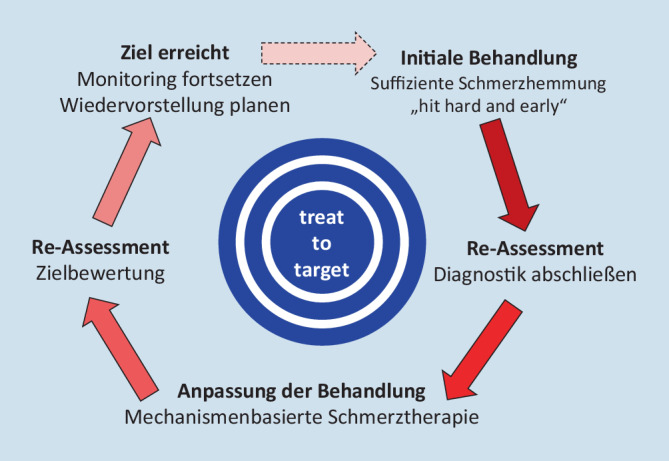

Angelehnt an die zielorientierte Therapieplanung und -führung in der Versorgung chronisch Rheumakranker [12, 75], plädiert der Autor bei Patienten mit (chronischem) degenerativem TRS orientiert an den differenzialtherapeutischen Grundsätzen der MM [55] unter Kenntnis von Techniken und Evidenzen der TLI (Tab. 5), für die Etablierung eines strukturierten mechanismenbasierten (Abb. 5; [52]) Therapiekonzeptes im Sinne des „treat to target“ (T2T) im ambulanten schmerztherapeutischen Versorgungsbereich (Abb. 6).

Der Autor plädiert für die Etablierung eines strukturierten mechanismenbasierten Therapiekonzeptes

Dies setzt eine konsequente (Primär‑)Diagnostik mit Schmerzanalyse unter der Prämisse, dass TRS, wenn strukturell-funktionell bedingt, immer spezifisch ist [2], voraus. Das heißt, die breite Anamnese (auch soziofamiliär zur Detektion von „yellow, red, blue, black flags“), mit strukturbezogener (Ausschluss‑)Diagnostik (klinisch-bildgebend) und funktioneller Differenzierung (nach MM-Grundsätzen, ggf. interventionelle Blocks), sollte am Ende zur Formulierung einer 3‑Ebenen-Diagnose als Voraussetzung zur mechanismenbasiert-zielorientierten hierarchischen Stufentherapie führen:

1. Initial wirksame symptomatische Schmerzbekämpfung („hit hard and early“):
  z. B. breite Neuraltherapie, peridurale TLI, analgetisch-antiphlogistische Infusion
2. idealerweise Detektierung und Stilllegung/Wegnahme des primären Nozigenerators (initiales T2T):
  z. B. gezielte, ggf. bildgestützte TLI (MM, wenn rein funktionelles Schmerzmuster)
3. Beseitigung residueller, reaktiver (sekundärer) funktioneller Schmerzmuster (myofasziale und/oder hypomobile Dysfunktion der LSIR):
  z. B. MM: Mobilisation, Manipulation, TLI: Neuraltherapie
4. bei (beginnender) Chronifizierung: mechanismenbasierte multimodale Schmerztherapie (prolongiertes T2T):
  Rückbau von Chronifizierungsvorgängen (Desensibilisierung): z. B. serielle TLI, medikamentöse Analgesie, ggf. Opiate (LONTS [36])
  Rekonditionierung der inhibitorischen Systeme: z. B. Akupunktur, Verhaltenstherapie
5. Muskuläre Rekonditionierung (Funktion, Balance) und Rekoordination der motorischen Systeme:
  z. B. aktive Krankengymnastik, Ergotherapie, Krankengymnastik am Gerät, ggf. ambulante/stationäre Rehabilitation
6. Regelhaftes Training der zurück- oder neugewonnenen Funktion als Rezidivprophylaxe (liegt in der Eigenverantwortung des Patienten)

## Schlussfolgerungen

**TRS**
- ist meistens spezifisch, d. h. er hat eine strukturelle oder funktionelle primäre Ursache. Nur selten ist er „nichtspezifisch“; oft mangelt es nur an der diagnostischen Sorgfalt und Konsequenz.
- bedarf einer zielgerichteten, ursachenspezifischen Therapie („treat to target“).
- bedarf initial einer raschen schmerzhemmenden Intervention („hit hard an early“).
- ist im postakuten Stadium selten mono-, sondern meistens multikausal (komplex, mehrere Schmerzgeneratoren): myofasziale/segmentale Dysfunktion ist oft sekundär, also Folge unterschiedlicher primärer Schmerzgeneratoren und dann bereits Ausdruck beginnender Chronifizierung.
- bedarf einer differenzierten Schmerzanalyse und mehrschichtiger Diagnosenstellung (3 Ebenen).
- setzt zur Behandlung vertiefte Kenntnisse in der Schmerzphysiologie voraus.
- bedarf bei Chronifizierung eines multimodalen Behandlungskonzeptes („Leader“: Spezieller Schmerztherapeut).

**TLI**
- relevantes diagnostisches Tool zur Spezifizierung von TRS
- relevantes initial therapeutisches Tool bei akuten, exazerbierten TRS („hits hard and early“)
- relevantes Tool für gezielte therapeutische Intervention am primären Schmerzgenerator („treats to target“)
- möglicher „gate opener“ für MM bei sekundärem funktionellem TRS
- relevante Methode zur Down-Regulierung chronisch-struktureller und -funktioneller TRS auf niedriges Niveau (Desensibilisierung: serielle Injektionen)
- hat bei qualifizierter Anwendung weniger unerwünschte Wirkungen im Vergleich zu dauerhafter medikamentöser Analgesie
- wichtige Alternative bei Ausschöpfung medikamentöser Analgesie (Unverträglichkeiten, Risikofaktoren)

Schlussfolgernd kann konstatiert werden, dass bei sinnvollem Einsatz von Techniken der TLI und MM im Sinne eines bi-/multimodalen Settings der degenerative (chronische) TRS rascher, gezielter und damit effektiver therapiert werden kann, auch wenn bzgl. der Therapieeffektivität bis dato v. a. die Mobilisationsmethoden der MM (und auch der Osteopathie), als auch die weichteil-neuraltherapeutischen, wie auch die landmarken-, sonographie- und CT-gestützten TLI einen schlechten bzw. fehlenden Evidenznachweis haben. Manipulative MM-Verfahren sind bis dato als mäßig effektiv evidenzbasiert bewertet. Lediglich bei röntgengestützten EPI-, FGI- und SIG-Infiltrationen sind unter Verwendung von LA-GK-Gemischen gute (bis moderate) Wirknachweise vorhanden. Gründe für die schlechten Evidenzlagen scheint es mehrere zu geben. Der Mangel an guten und das Vorhandensein von schlechte Studienqualitäten mit fehlender Bildung an RCT liegt einerseits daran, dass v. a. in der MM die Bildung von „Sham“-/Placebogruppen methodenbedingt schwierig ist. Anderseits sind bisherige (Sub‑)Gruppierungen gescheitert oder mangelhaft aufgrund einer unpräzisen Diagnostik (Mischdiagnosen) bei variabler Symptomatik bzw. Multifaktorialität möglicher Schmerzursachen und uneinheitlicher Terminologie, aber auch aufgrund indifferenter Therapiesettings und variabler Therapeutenqualität.

Es besteht die Notwendigkeit der Etablierung spezifischer subgruppenbasierter RCT und Metaanalysen. Voraussetzung zu Bildung solcher Gruppen wäre jedoch eine präzise, konsequente standardisierte Diagnostik auf Basis einer weiterzuführenden einheitlichen Terminologie, wenngleich diesbezüglich in den letzten Jahren durch das Engagement entsprechender Fachorganisationen (IGOST, DGMM, SAMM, DAAO) schon viel erreicht wurde.

## Fazit für die Praxis

- Ohne vertiefte Kenntnis (patho-)physiologischer neuronaler Schmerzentstehungs- und -verarbeitungsmechanismen ist eine gezielte und effektive Behandlung (sub-)chronischer tiefer Rückenschmerzen (TRS) nicht möglich.
- Werden konservative/interventionelle Methoden, wie Manuelle Medizin (MM) und therapeutische Lokalinfiltration (TLI), lediglich symptomatisch orientiert bezüglich sekundärer (funktioneller) Schmerzgeneratoren eingesetzt, besteht die Gefahr der (iatrogenen) Schmerzchronifizierung; dies meist aufgrund insuffizienter Primär- oder Folgediagnostik (Re-Assessment).
- Die Detektion der primären Schmerzgeneratoren ist Voraussetzung für eine erfolgreiche mechanismenbasierte Therapie beim spezifischen TRS.
- MM und TLI sind empiriebasierte und partiell evidenzbasierte, wirkungsvolle Instrumente in der Behandlung akuter und chronischer spezifischer TRS, welche bei mechanismenbasiert stadiengerechtem, gezielt synergistischem bi-/multimodalem Einsatz eine effektive Therapie und Chronifizierungsprophylaxe gewährleisten können.
- Monomodale Therapiekonzepte werden dem postakuten, rezidivierenden TRS selten gerecht und bergen eine hohe Gefahr der Chronifizierung.

## Abkürzungen

ACLS: „Advanced cardiac life support“
ASIPP: „American Society of Interventional Pain Physicians“
BÄK: Bundesärztekammer
BMI: Body-Mass-Index
Btx: Botulinumtoxin
BV: Bildverstärker (Bildwandler)
CGRP: „Calcitonin-gene related peptide“
DAAO: Deutsch-Amerikanische Akademie für Osteopathie e. V.
DGAI: Deutsche Gesellschaft für Anästhesiologie und Intensivmedizin e. V.
DGMM: Deutsche Gesellschaft für Manuelle Medizin e. V.
EPI: Epidurale Injektion
FG: Facettengelenk
FGI: Interventionen am Facettengelenk
FGS: Facetten(gelenk)syndrom
GK: Glukokortikoide
HVLA: „High velocitiy low amplitude“
IDT: Intradiskale Interventionen
IGOST: Interdisziplinäre Gesellschaft für orthopädische/unfallchirurgische und allgemeine Schmerztherapie e. V.
IL: Interleukin
INR: International Normalized Ratio
IVR: Intervertebralraum
KHK: Koronare Herzkrankheit
LA: Lokalanästhetikum
LSIR: Lumbosakroiliakalregion
LSPA: Lumbale Spinalnervanalgesie
LWS: Lendenwirbelsäule
MIP: Mobilität, Irritation, Provokation
MM: Manuelle Medizin
MPSS: Mainzer Stadienmodell der Schmerz-Chronifizierung
PRP: „Platteled enriched plasma“
PRT: Periradikuläre Therapie
RFN: Radiofrequenz-Neurotomie
ROM: „Range of motion“
SAMM: Schweizerische Ärztegesellschaft für Manuelle Medizin
SIG: Sakroiliakalgelenk
SIPS: Spina iliaca posterior superior
SNR: „Signal-noise-ratio“
T2T: „Treat to target“
TLA: Therapeutische Lokalanästhesie
TLI: Therapeutische Lokalinfiltration/-injektion
TNF: Tumornekrosefaktor
TRS: Tiefer Rückenschmerz
VAS: Visuelle Analogskala
WDR: „Wide dynamic range neuron“
ZNS: Zentrales Nervensystem
